# Elucidating the Visual Snow Spectrum: A Latent Class Analysis Study

**DOI:** 10.1155/2024/5517169

**Published:** 2024-01-19

**Authors:** Amy Claire Thompson, Patrick T. Goodbourn, Jason D. Forte

**Affiliations:** Melbourne School of Psychological Sciences, University of Melbourne, Parkville, Australia

## Abstract

**Objective:**

People with visual snow syndrome (VSS) experience a range of perceptual phenomena, in addition to visual snow (VS; flickering pinpricks of light throughout the visual field). We investigated the patterns of perceptual phenomena associated with VSS in a large sample of people without prior knowledge of VSS or its associated symptoms. *Methods and Measures*. Two thousand participants completed a screening questionnaire assessing the frequency and severity of perceptual phenomena associated with VSS. We used latent class analysis (LCA), a clustering technique which identifies qualitatively different subgroups within a given population, to investigate whether the presence (or absence) of VS impacted class structure.

**Results:**

Of 1,846 participants included for analysis, 41.92% experienced VS some of the time, including 4.49% who had VSS without prior knowledge. The mean number of perceptual phenomena experienced was 2.03. Optimal four-class LCA solutions did not substantially differ whether VS was included in the model; instead, classes differed in the frequency and total number of symptoms experienced. *Discussion*. Our results suggest that the perceptual phenomena associated with VSS are likely to be common in the general population and do not necessarily indicate an underlying pathology. We also showed that visual snow itself does not explain the presence of other perceptual phenomena.

## 1. Introduction

Visual snow (VS) is a perceptual phenomenon characterized by persistent flickering noise in the visual field. It is often described as being like a sensation of pixelation, or the “snow” of an out-of-tune analogue television, from which it takes its name. It is also the primary perceptual experience associated with visual snow syndrome (VSS). People with VSS experience visual snow constantly, along with at least two additional perceptual phenomena from a list including nyctalopia (poor night vision), photophobia (pain or discomfort in bright light), palinopsia (trailing after-images), and a range of enhanced entoptic phenomena [1, 2]. VSS can be either lifelong or acquired. Research suggests that as many as 36% of people with VSS have experienced it for as long as they can remember [3]. However, many people with lifelong VSS do not realize their perceptual experience is unusual and thus do not pursue a diagnosis. Consequently, this group is often not accounted for in research findings.

Research has often described VSS as a spectrum-based disorder because it is associated with a range of perceptual experiences and levels of severity [4, 5]. People with *confirmed* (i.e., medically or self-diagnosed) VSS experience a variety of different impacts, from minor annoyance or fascination, to major disruptions to quality of life [3, 6]. The number of additional symptoms experienced, and the frequency with which these are experienced, also varies substantially between patients [4, 7]. There is also growing anecdotal evidence from online support groups associated with VSS that some people self-describe as having VSS despite never or rarely experiencing visual snow. These people tend to experience a wide range of symptoms associated with VSS but do not meet the current diagnostic criteria, which require the continuous experience of visual snow [8].

Research to date has not investigated whether the visual snow spectrum might extend beyond the existing diagnostic criteria, to include a range of normal perceptual experiences in the general population. Several recent studies have provided evidence that both visual snow and VSS are somewhat common in the general population; around 40% of people experience visual snow at least some of the time, while a 2020 prevalence estimate shows that 2.2% of people meet the diagnostic criteria for VSS without prior knowledge [6, 7, 9]. These studies suggest that both lifelong and undiagnosed VSS may be highly prevalent and that VSS may not always have noticable impacts on the people who experience it. Given the large proportion of people who report experiencing visual snow some of the time—and the fact that most other VSS symptoms are experienced by most people from time to time—it seems feasible that an extended visual snow spectrum might exist. Identifying and understanding population-level experiences of perceptual phenomena associated with VSS may be instructive in understanding how a wide range of perceptual experiences, with a variety of known and unknown mechanisms, cooccur so consistently in people with VSS.

## 2. The Present Study

The present study investigated the extent to which perceptual experiences associated with VSS cooccur in the general population. We used latent class analysis (LCA) to investigate subgroups of perceptual experience, based on patterns of presence, frequency, and cooccurrence of the perceptual phenomena that make up the VSS diagnostic criteria. Given anecdotal evidence suggesting many people experience a broad range of additional phenomena associated with VSS in the absence of visual snow, we also investigated whether the presence of visual snow was essential to model classification, as it is to the VSS diagnostic criteria.

## 3. Methods

This study was approved by The University of Melbourne Human Research Ethics Committee and was conducted in accordance with the Declarations of Helsinki. Participants provided informed consent via an online form prior to the commencement of the study.

### 3.1. Participants

We recruited a sample of 2,000 naïve participants via Amazon Mechanical Turk (MTurk). Participants were required to be 18 years of age or older and to be fluent in English. All participants were reimbursed US $1 for their participation. The study was advertised to 1,000 participants at a time across two dates in 2022 to ensure that it remained near the top of lists of available “tasks” on MTurk. Participants who engaged in the study when it was first advertised were prevented from participating again. The study was advertised under the title “Answer a survey about your vision (5-20 mins).” Aside from practical information relating to payment, inclusion criteria, and the time we expected the task to take, the following text was used to advertise the study:

*You will be asked about some perceptual experiences you may or may not have had, and about some of your medical history, because conditions such as migraine can impact visual perception.*

It is possible that recruiting participants via this method introduced a degree of selection bias, as participants with particular interest in their visual experience (due to concern, fascination, or otherwise) may have been more likely to choose to participate. We did not attempt to verify that our participants were a representative sample of the general population, but rather chose to use the largest sample we reasonably could.

#### 3.1.1. Response Screening

Participants were screened to assess for responses from bots, responses which did not meet inclusion criteria, and bad-faith responses. In addition to reCAPTCHA and a series of standard attention checks, all free-text answers were assessed to determine if responses were reasonable. In total, 92.3% of participants (*n* = 1,846) were considered valid respondents and included for analysis.

### 3.2. Measures

Participants completed a screening questionnaire assessing the presence, frequency, and perceived impact of perceptual phenomena currently included in the VSS diagnostic criteria. The questionnaire was adapted from the work of Kondziella et al. [9]. The only substantive change made was that participants who stated they experienced a given perceptual phenomenon were asked to rate how often they experienced this phenomenon and how often they felt their life was impacted by the phenomenon (daily, weekly, monthly, several times a year, or yearly). All language describing perceptual experiences was repeated exactly from Kondziella et al.'s work.

This paper represents the primary analysis of these data. However, data collection for this study formed part of a broader project, and participants who met the criteria for VSS engaged with additional scale-based measures related to sensory sensitization, which are not reported here.

#### 3.2.1. Diagnostic Categorization

Participants were categorized as experiencing visual snow, VSS, and migraine with or without aura in accordance with the categorization process described by Thompson et al. [7].

### 3.3. Statistical Analyses

#### 3.3.1. Software

All analyses were conducted using R 4.0.3, and all graphics were generated using ggplot2 [10, 11]. Data preprocessing and scale scoring were conducted using the psych package [12]. Latent class analysis was conducted using poLCA, and graphs for latent class analysis were generated using open-access code sourced from GitHub [13, 14]. Cohen's weighted Kappa was calculated using vcd [15]. All other analyses, including demographic between-group comparisons, were conducted using base R and its associated stat package.

#### 3.3.2. Latent Class Analysis

Latent class analysis is a technique which identifies qualitatively different subgroups within a population based on shared characteristics. Its core assumption is that membership of unobserved classes can explain patterns of scores across survey questions or scales [16]. We sought to identify classes of participants based on experiences of perceptual phenomena commonly described in the VSS literature. Phenomena included for analysis were visual snow, photophobia, nyctalopia, self-light of the eyes, blue-field entoptic phenomenon, halos, palinopsia, and excessive floaters.

We began by estimating a one-class model and added classes up to eight. Starting values were determined randomly. To ensure model stability, each model was estimated 100 times and the model with the lowest log-likelihood was used. All models had suitable entropy (>0.8) [16]. For each model, we examined fit based on (a) the Bayesian information criterion (BIC), (b) sample-size adjusted BIC (SABIC), (c) the Akaike information criterion (AIC), and (d) *G*^2^, the likelihood ratio statistic. In all cases, lower values indicate better model fit. Because there was a discrepancy between fit statistics, the final model selection was made based on balancing good fit with a model which most logically explained the data [16].

### 3.4. Data Sharing

The data that support the findings of this study are available from the corresponding author upon reasonable request.

## 4. Results

Of 1,846 participants included for analysis, 774 (41.92%) experienced visual snow at least some of the time, including 83 (4.49%) who met the International Classification of Headache Disorders (ICHD) criteria for visual snow syndrome (without prior knowledge). Hallucinogen persisting perceptual disorder could not be ruled out in 15 cases of VSS, where drug use immediately preceded the onset of perceptual experiences. These participants are not included in the VSS count.

**Figure 1:**
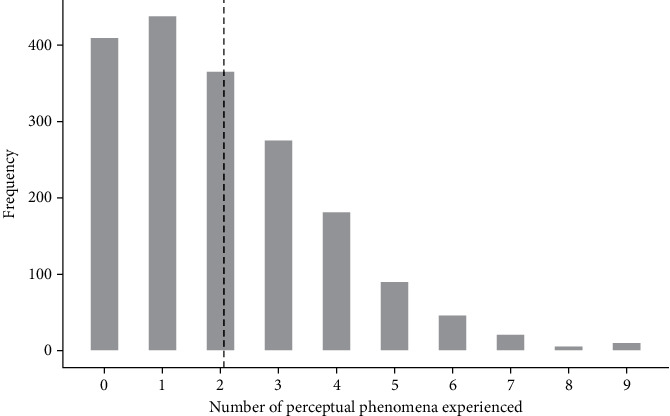
Frequency chart showing the number of perceptual phenomena experienced by participants. Note. Dotted line indicates the mean number of perceptual phenomena experienced.

Participants' mean age was 38.34 years, and the range was 18 to 80 years. 1,084 participants were identified as male, 746 identified as female, 7 identified as nonbinary, and 9 preferred not to say. The mean number of perceptual phenomena experienced (including visual snow) was 2.03 (SD = 1.79). A summary of sample characteristics is presented in Table 1, and Figure 1 presents the frequency distribution of the number of perceptual phenomena experienced in our sample.

### 4.1. Between-Group Comparisons

We investigated whether there were differences between participants with visual snow, VSS, cases of VSS where HPPD could not be ruled out, and those without visual snow.  Table 2 presents data pertaining to perceptual phenomena and comorbid conditions per group.

First, we investigated whether there were differences in the number of perceptual phenomena experienced between participants in each category. A Kruskal-Wallis *H* test showed significant differences, *χ*^2^(3) = 537.67, *p* < 0.001, *η*^2^ = 0.29. Post hoc, Holm-corrected Wilcoxon pairwise tests revealed no significant difference between participants where HPPD could not be ruled out and those with VSS (*p* = 0.55). However, all other combinations of variables showed significant differences at the *p* < 0.001 level.

Next, we used a series of chi-squared tests of independence to investigate whether there were associations between the above-described categories and relevant medical history. The results for migraine category were significant, *χ*^2^(6) = 413.37, *p* < 0.001, Cramer's *V* = 0.335; as were the results for tinnitus presence or absence, *χ*^2^(3) = 21.594, *p* < 0.001, *V* = 0.108; and history of recreational drug use, *χ*^2^(3) = 46.054, *p* < 0.001, *V* = 0.158.

### 4.2. Latent Class Analysis

Our first latent class model included data on the presence and frequency of all perceptual phenomena associated with VSS. Based on the various fit statistics presented in Table 3, we selected a four-class model as the most parsimonious fit for the data. The inconsistencies in fit statistics are not unusual in LCA, and Weller et al. suggest that in this scenario, the SABIC should be relied upon in decision-making [16].

The four classes in our model include (1) a *high-intensity* class, comprising people who experience many perceptual phenomena associated with VSS, with a high degree of frequency; (2) and (3) two *medium-intensity* classes, comprising people who experience some perceptual phenomena associated with VSS, with less frequency in class 3 than class 2; and (4) a *low-intensity* class, comprising people who rarely experience phenomena associated with VSS.

**Figure 2:**
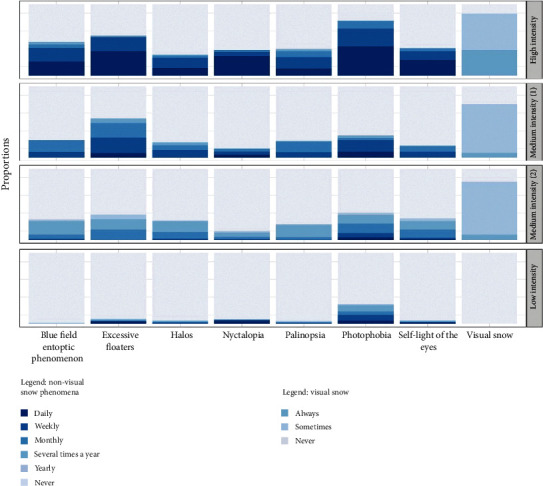
A four-class model including all perceptual experiences associated with VSS as predictors.

The composition of each of our four classes is illustrated in Figure 2. Here, each box represents a different class. Along the *x*-axis are perceptual phenomena. The proportion of each bar that is shaded in each color indicates the conditional probability that someone who experiences that perceptual phenomenon with that frequency will be included in each class. Importantly, a different scale was used to assess the frequency of visual snow as this question was taken directly from the work of Kondziella et al. [9].

Initial estimates of class population shares and final participant class allocations based on posterior probabilities differed slightly. Based on final class allocations, the *high-intensity* class comprised 173 (9.37%) participants. This class most closely matches VSS; participants in this class experience many perceptual phenomena daily. However, not all participants in this class experience visual snow, and many of those who do experience visual snow do not experience it continuously. The fact that half (*n* = 937, 50.76%) of participants fell in the *low-intensity* class and that many of these participants experienced some VSS-related phenomena (albeit rarely) shows that the experience of perceptual phenomena associated with VSS is common in the general population. The two medium-intensity categories comprised 343 (18.58%) and 393 (21.29%) participants, respectively.

We compared the model's classifications against the VSS diagnostic criteria. In total, 9 participants who met the diagnostic criteria for VSS were included in the *high-intensity* class, along with 104 participants who did not experience VS, and 59 who experienced visual snow in the absence of the full syndrome. Meanwhile, 47 participants who met the VSS diagnostic criteria were in the first medium-intensity class; 15 were in the second medium-intensity class; and 12 were included in the *low-intensity* category. This indicates that simply meeting the VSS diagnostic criteria does not necessarily mean experiencing a wide range of symptoms regularly.

#### 4.2.1. Is Visual Snow Essential to Model Classification?

**Figure 3:**
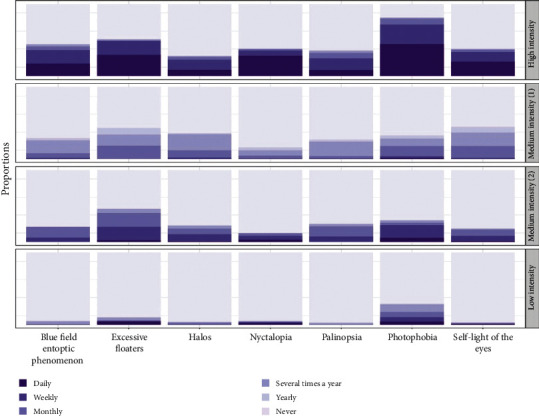
A four-class model excluding visual snow as a predictor.

To determine if visual snow is essential to model classification, as it is to the VSS diagnostic criteria, we conducted LCA with the visual snow variable removed as a predictor. Once again, we selected a four-class model as the most parsimonious fit for the data (Table 4); and broadly, the LCA model in the absence of VS was like that in the presence of VS.  Figure 3 presents the full results of this model, in the same format as for the previous model.

Based on posterior probabilities, the proportions of participants in each class in this model differed slightly from those of the model including visual snow. The *high-intensity* class gained 20 participants to a total of 193 (10.4%). However, the two *medium-intensity* classes and the *low*-*intensity* class saw shifts in their relative sizes. *Medium-intensity* (1) comprised 185 (10.02%) participants and *medium-intensity class* (2) comprised 383 (20.75%) participants. Finally, the *low*-intensity class grew to 1,085 (58.77%) participants.

To investigate whether these changes in class composition indicated that the model had changed substantially in the absence of visual snow, we calculated Cohen's weighted Kappa to determine the degree of correspondence between the two models: *κ* = 0.75, 95% CI (0.72, 0.79), *p* > 0.05. This indicates substantial agreement between the two models. The confusion matrix presented in Table 5 shows that most changes to class allocations between models occurred when participants who had been in *medium intensity* (2) in the model including visual snow were allocated to *medium intensity* (1) in the model excluding visual snow. Participants who had been in the *high-intensity* and *low-intensity* classes in the model including visual snow were almost all still in those classes in the model excluding visual snow. Overall, this suggests that the model was relatively robust even when visual snow was removed.

## 5. Discussion

In this study, we used latent class analysis to investigate population distributions of perceptual phenomena associated with visual snow syndrome (VSS). We demonstrated that perceptual phenomena associated with VSS are likely to be common in the general population; approximately half of the participants experienced some perceptual phenomena associated with VSS, some of the time. Our results also indicate the presence of a visual snow spectrum, which includes perceptual experiences that extend beyond the existing diagnostic criteria. Using all perceptual phenomena associated with VSS as predictors, we identified a four-class LCA model as the most parsimonious fit for our data. The four classes were based on subgroups of participants whose perceptual experiences increased in number and frequency across classes, resembling a spectrum.

We also showed that visual snow itself was not essential to model classification. When we removed visual snow as a predictor variable, a four-class model remained the most parsimonious fit for the data, and the four classes were similar in size and composition. Once participants were allocated to classes based on posterior probabilities, we used Cohen's weighted Kappa as a measure of agreement between the two models, with the result indicating substantial agreement. Few participants moved between the *high-* and *low-intensity* classes when visual snow was removed from the model. This suggests that visual snow itself may not be key to explaining patterns of perceptual experiences and may not be the defining feature of the spectrum we identified. However, it is important to note that visual snow was measured using a different scale than the other perceptual experiences we assessed; this may have artificially diminished its importance in the model.

### 5.1. Is Visual Snow Normal?

Our results show some similarities to existing work in the field and indicate that both visual snow and other perceptual phenomena associated with VSS are common perceptual experiences. For example, the percentage of our participants who experienced visual snow at least some of the time is remarkably similar to the findings of Costa et al., whose work shows that 44% of people experience visual snow at least 10% of the time [6]. Meanwhile, 41.92% of our participants reported experiencing visual snow some of the time. These estimates also correspond with our previous work [7]. Together, they provide further evidence that visual snow is a common perceptual experience for which most people do not require clinical attention.

Our results also correspond with the only previous LCA conducted on data related to VSS. In a study of 1,060 participants with confirmed VSS, Puledda et al. demonstrated that additional symptoms of VSS do not present in specific combinations, but that floaters, palinopsia, and photophobia are “almost invariably present.” [4]. This matches well with our own findings. Our model did not identify classes based on cooccurrence of specific symptoms, but rather based on the total number and frequency of symptoms experienced. Floaters and photophobia were also the most common perceptual phenomena in our sample. However, while Puledda et al.'s latent class models provided support for the current VSS diagnostic criteria [7], our results indicate that the range of perceptual experiences associated with VSS goes beyond the existing diagnostic criteria. Almost half of our participants fell in our first three classes, suggesting that it is common to experience perceptual phenomena with some degree of regularity. While some people find these phenomena impactful in their day-to-day lives, the presence of the phenomena themselves may not necessarily indicate an underlying clinical pathology.

Like other work showing visual snow spectrum phenomena are common in the general population, this study was conducted online via population screening. To date, work of this kind has not been validated against traditional diagnostic techniques, and it is possible that these studies have captured less impactful perceptual experiences than those identified by clinical diagnosis. However, there is as yet no specific test for VSS and clinical diagnosis is by exclusion. It is also important to note that recruitment via online tools involves participants self-selecting into research based on brief descriptions of what is being studied; this may lead to self-selection bias, with participants who have unusual perceptual experiences being more likely to engage with studies like this one. However, as three separate studies (including this one) have found a visual snow prevalence of around 40% using various online screening techniques, we are confident that the perceptual experiences we identified are genuine. Whether they are the same as those identified in people with confirmed VSS, and whether our model findings will generalize to populations with confirmed VSS, remains to be seen. To this end, future research should address whether patterns of perceptual experience in confirmed VSS are defined by visual snow.

### 5.2. Clinical Implications

We have shown that a population-level visual snow spectrum likely exists, and that visual snow spectrum perceptual experiences are common in the general population. Our results indicate that the number of perceptual phenomena experienced may move someone closer to meeting the diagnostic criteria for VSS but does not necessarily indicate the impact on day-to-day life, as our participants form a nonclinical sample. As there is currently no objective measure of VSS severity, clinicians and researchers must evaluate people's own descriptions of the impact of their perceptual experiences. Our work, along with that of Costa et al., shows that it is possible to experience numerous visual snow spectrum phenomena in the absence of either visual snow itself, and in the absence of negative impacts [6]. As such, the question remains: what causes visual phenomena to be—or become—distressing?

At present, the most promising solution to this problem lies in the affective response to perceptual experiences. Certain mental health conditions are commonly associated with VSS, and it has been argued that they may be inherent to the VSS phenotype [3]. For example, in participants with confirmed VSS, Solly et al. found clinical levels of depression and anxiety, the presence of depersonalization and derealization, and sleep problems and fatigue [3]. It is possible that this psychopathology is not inherent to VSS, but rather that it is what elevates perceptual phenomena to be impactful on daily life. Most importantly, whether these conditions are inherent to VSS or regularly comorbid with it, they all have existing, recommended treatment protocols, and treating comorbid mental health conditions has been effective in reducing symptom impact in related conditions such as tinnitus [17]. While psychological and behavioral interventions may not alter perceptual phenomena themselves, they may provide some relief for certain patients.

Recently, Wong et al. have published data showing that mindfulness-based cognitive therapy (MBCT) can be effective in relieving the symptoms of VSS [18]. The results of this study showed subjective symptom improvement after MBCT in a small sample of people with clinically diagnosed VSS and demonstrated that subjective improvements were associated with changes in fMRI results. The researchers found that, three months after intervention, fMRI results showed alterations in the functional connectivity of the visual network, with changes noticeable in extra-striate regions of the occipital cortex, areas of the cerebellum which are related to visual processing and attention, and the posterior hub of the default mode network. This study both provides evidence that the subjective experience of VSS can be improved through psychological interventions and demonstrates that such interventions can have functional implications, potentially impacting both subjective and objective experience. Further work investigating mechanisms which cause visual snow spectrum phenomena to become distressing will be important—as will work investigating additional psychological interventions for symptom relief in people with severe presentations.

A limitation of the present study is that we did not collect data pertaining to psychopathology in our sample. As such, we can only speculate about the role of comorbid mental health conditions in the perceived severity of VSS, based on existing literature which indicates a connection between mental health conditions and VSS. Future research should consider comparing psychopathology and mental health symptoms in samples of naïve participants who meet the VSS diagnostic criteria and in people with clinical diagnoses (or self-diagnoses).

## 6. Conclusion

In this study, we demonstrated that perceptual phenomena associated with VSS are common in the general population and that visual snow is not key to explaining the presence of these phenomena. We also identified a spectrum of perceptual experiences associated with VSS, with people who experience numerous perceptual phenomena often at one extreme, and people who rarely experience any perceptual phenomena at the other. Our results indicate that visual snow spectrum perceptual experiences are common and do not necessarily indicate underlying pathology. In the absence of objective measures of VSS severity, it seems that the same number and frequency of VSS symptoms can be distressing to some people, while others can ignore them entirely. Future research should investigate whether visual snow is key to explaining the perceptual experiences of people with confirmed VSS and to addressing whether psychological interventions are effective in relieving the distress caused by VSS.

## Figures and Tables

**Table 1:** Summary of sample characteristics.

	Complete sample	Participants without VS	Participants with VS	Participants with VSS
*N*	1,846	1,072	774	83
Mean age (SD)	38.34 (12.03)	37.79 (12.76)	36.32 (10.62)	37.64 (12.26)
Gender (male)	1,084 (58.72%)	649 (60.54%)	435 (56.20%)	45 (54.21%)
Gender (female)	746 (40.41%)	413 (38.53%)	333 (43.02%)	38 (45.79%)
Gender (nonbinary)	7 (0.38%)	4 (0.37%)	3 (0.39%)	0 (0.00%)
Gender (prefer not to say)	9 (0.49%)	6 (0.56%)	3 (0.39%)	0 (0.00%)
Relevant medical history				
Probable migraine (no aura)	260 (14.08%)	99 (9.24%)	75 (9.69%)	10 (12.05%)
Probable migraine (aura)	500 (27.09%)	185 (17.25%)	401 (51.81%)	42 (50.60%)
Tinnitus	402 (21.78%)	207 (19.31%)	195 (25.20%)	33 (39.75%)
Drug history	668 (36.19%)	339 (31.62%)	329 (42.51%)	30 (36.14%)
Perceptual phenomena associated with VSS				
Palinopsia	281 (15.22%)	81 (7.56%)	200 (25.84%)	33 (39.76%)
Excessive floaters	519 (28.11%)	148 (13.81%)	371 (47.93%)	60 (72.29%)
Blue field entoptic phenomenon (BFEP)	307 (16.63%)	41 (3.82%)	266 (34.37%)	44 (53.01%)
Halos	288 (15.60%)	86 (8.02%)	202 (26.10%)	25 (30.12%)
Self-light of the eyes	300 (16.25%)	92 (8.58%)	208 (26.87%)	31 (37.35%)
Photophobia	658 (35.64%)	310 (28.92%)	348 (44.96%)	50 (60.24%)
Nyctalopia	222 (12.03%)	82 (7.65%)	140 (18.10%)	19 (22.89%)

Note. The participants with visual snow here also include the participants with VSS and participants with possible HPPD. Participants who had possible HPPD, i.e., met the criteria for VSS and who had a history of drug use which was immediately prior to the onset of their perceptual phenomena, are not included in the VSS category.

**Table 2:** Group means for perceptual phenomena and comorbid conditions.

	Participants without VS	Participants with VS	Participants with VSS	Participants with possible HPPD
*N*	1,072	676	83	15
Perceptual phenomena experienced (mean (SD))	0.97 (1.02)	3.33 (1.52)	4.55 (1.52)	4.80 (1.61)
Migraine category				
Probable migraine (with aura)	99 (9.24%)	351 (51.92%)	42 (50.60%)	8 (53.33%)
Probable migraine (no aura)	185 (17.26%)	63 (9.32%)	10 (12.05%)	2 (13.33%)
No migraine	788 (23.51%)	262 (24.44%)	31 (37.35%)	5 (33.33%)
Tinnitus category				
Tinnitus	207 (19.31%)	157 (23.22%)	33 (39.76%)	5 (33.33%)
No tinnitus	865 (80.69%)	519 (76.78%)	50 (60.24%)	10 (66.66%)
Recreational drug use history				
Has disclosed drug use history	339 (31.62%)	284 (42.01%)	33 (39.76%)	15 (100%)
Has not disclosed drug use history	733 (68.38%)	392 (57.99%)	50 (60.24%)	0

Note. Unless otherwise specified, data are counts (percentages). The participants with visual snow here exclude the participants with VSS.

**Table 3:** Evaluating class solutions for a model including all predictors.

Classes	Degrees of freedom	Log-likelihood	BIC	SABIC	AIC	*G* ^2^ likelihood ratio statistic	Entropy
1	1809	-11576.13	23430.53	23312.98	23226.26	5572.40	6.271
2	1771	-11004.04	22527.13	22333.85	22158.07	4428.21	5.971
3	1733	-10739.56	**22328.98**	21969.98	21705.13	3899.27	5.838
4	1695	-10656.53	22448.69	**21968.97**	21615.05	3733.19	5.781
5	1657	-10595.37	22612.18	22011.73	21568.75	3610.89	5.748
6	1619	-10542.09	22791.40	22070.23	21538.18	3504.32	5.721
7	1581	-10495.78	22984.57	22142.68	21521.57	3411.71	5.693
8	1581	**-10453.59**	23185.97	22223.35	**21513.17**	**3327.31**	5.668

Note. The lowest values for each fit statistic are in bold.

**Table 4:** Evaluating class solutions for a model excluding visual snow.

Classes	Degrees of freedom	Log-likelihood	BIC	SABIC	AIC	*G* ^2^ likelihood ratio statistic	Entropy
2	1775	-9633.18	**19800.33**	19574.76	19408.35	3111.82	5.232
3	1739	-9504.18	19813.09	19473.15	19222.36	2853.83	5.166
4	1739	-9424.37	19924.22	**19469.91**	19134.74	2694.21	5.166
5	1667	-9375.57	20097.36	19528.68	19109.14	2596.62	5.085
6	1631	-9330.80	20278.56	19595.52	19091.60	2507.07	5.058
7	1595	**-9288.74**	20465.21	19667.79	**19079.49**	**2422.96**	5.058

Note. The lowest values for each fit statistic are in bold.

**Table 5:** Confusion matrix comparing participant posterior allocations between latent class models including and excluding visual snow.

Visual snow included	Visual snow excluded			
	High intensity	Medium (1)	Medium (2)	Low intensity
High intensity	154	17	0	2
Medium (1)	24	845	12	56
Medium (2)	1	192	173	27
Low intensity	14	31	0	298

## Data Availability

The data that support the findings of this study are available from the corresponding author upon reasonable request.
